# Bottleneck Size-Dependent Changes in the Genetic Diversity and Specific Growth Rate of a Rotavirus A Strain

**DOI:** 10.1128/JVI.02083-19

**Published:** 2020-05-04

**Authors:** Syun-suke Kadoya, Syun-ichi Urayama, Takuro Nunoura, Miho Hirai, Yoshihiro Takaki, Masaaki Kitajima, Toyoko Nakagomi, Osamu Nakagomi, Satoshi Okabe, Osamu Nishimura, Daisuke Sano

**Affiliations:** a Department of Civil and Environmental Engineering, Graduate School of Engineering, Tohoku University, Aoba-ku, Sendai, Miyagi, Japan; b Graduate School of Life and Environmental Sciences, University of Tsukuba, Tsukuba, Ibaraki, Japan; c Research Center for Bioscience and Nanoscience (CeBN), Japan Agency for Marine-Earth Science and Technology (JAMSTEC), Yokosuka, Kanagawa, Japan; d Super-cutting-edge Grand and Advanced Research (SUGAR) Program, Japan Agency for Marine-Earth Science and Technology (JAMSTEC), Yokosuka, Kanagawa, Japan; e Division of Environmental Engineering, Faculty of Engineering, Hokkaido University, Sapporo, Hokkaido, Japan; f Department of Molecular Microbiology and Immunology, Nagasaki University, Nagasaki, Japan; g Department of Environmental Studies, Tohoku University, Aoba-ku, Sendai, Miyagi, Japan; Cornell University

**Keywords:** NGS, bottleneck effect, genetic diversity, population genetics, quasispecies, rotavirus, sequence space, specific growth rate

## Abstract

RNA viruses form a dynamic distribution of mutant swarms (termed “quasispecies”) due to the accumulation of mutations in the viral genome. The genetic diversity of a viral population is affected by several factors, including a bottleneck effect. Human-to-human transmission exemplifies a bottleneck effect, in that only part of a viral population can reach the next susceptible hosts. In the present study, two lineages of the rhesus rotavirus (RRV) strain of rotavirus A were serially passaged five times at a multiplicity of infection (MOI) of 0.1 or 0.001, and three phenotypes (infectious titer, cell binding ability, and specific growth rate) were used to evaluate the impact of a bottleneck effect on the RRV population. The specific growth rate values of lineages passaged under the stronger bottleneck (MOI of 0.001) were higher after five passages. The nucleotide diversity also increased, which indicated that the mutant swarms of the lineages under the stronger bottleneck effect were expanded through the serial passages. The random distribution of synonymous and nonsynonymous substitutions on rotavirus genome segments indicated that almost all mutations were selectively neutral. Simple simulations revealed that the presence of minor mutants could influence the specific growth rate of a population in a mutant frequency-dependent manner. These results indicate a stronger bottleneck effect can create more sequence spaces for minor sequences.

**IMPORTANCE** In this study, we investigated a bottleneck effect on an RRV population that may drastically affect the viral population structure. RRV populations were serially passaged under two levels of a bottleneck effect, which exemplified human-to-human transmission. As a result, the genetic diversity and specific growth rate of RRV populations increased under the stronger bottleneck effect, which implied that a bottleneck created a new space in a population for minor mutants originally existing in a hidden layer, which includes minor mutations that cannot be distinguished from a sequencing error. The results of this study suggest that the genetic drift caused by a bottleneck in human-to-human transmission explains the random appearance of new genetic lineages causing viral outbreaks, which can be expected according to molecular epidemiology using next-generation sequencing in which the viral genetic diversity within a viral population is investigated.

## Abstract (precis)

In this study, we investigated a bottleneck effect on an RRV population that may drastically affect the viral population structure. RRV populations were serially passaged under two levels of a bottleneck effect, which exemplified human-to-human transmission. As a result, the genetic diversity and specific growth rate of RRV populations increased under the stronger bottleneck effect, which implied that a bottleneck created a new space in a population for minor mutants originally existing in a hidden layer, which includes minor mutations that cannot be distinguished from a sequencing error. The results of this study suggest that the genetic drift caused by a bottleneck in human-to-human transmission explains the random appearance of new genetic lineages causing viral outbreaks, which can be expected according to molecular epidemiology using next-generation sequencing in which the viral genetic diversity within a viral population is investigated.

## INTRODUCTION

RNA viruses form a dynamically distributed mutant swarm, termed a “quasispecies” (1, 2), because the proofreading function is absent from RNA-dependent RNA polymerase (RdRp). A mutant swarm, as a cloud in sequence space that may accommodate all sequences, is composed of one (or a few) master sequence that is temporally advantageous in the current environment as well as low-frequency minor sequences. Such dynamic genetic diversity of RNA viruses is advantageous for better adaptation to a given environment. For example, the replicative ability of West Nile virus has increased with an expansion of the size of a mutant swarm (3), and minor mutants of rabies virus have affected adaptation to a new host even when a master sequence was not replaced (4). These examples demonstrate that the increase of genetic diversity can provide phenotypic benefits to a virus population.

Infinite incrementation of genetic diversity within an RNA virus population is restricted by external factors in the environment, such as natural selection (advantageous genes are fixed in the population or deleterious mutations are removed from the population) and bottleneck effect (genes accidentally fix in and disappear from a population when the size of that population decreases temporarily; the subsequent random change of population structure is called genetic drift) (5). Such a bottleneck effect may impact the viral population structure, and transmission from host to host (even within a host) can work on a virus population as a bottleneck effect (6, 7). For example, Vignuzzi et al. reported that the diversity of a mutant swarm determined the pathogenesis of poliovirus, as the reduced diversity of poliovirus led to the loss of neurotropism and attenuated pathogenicity (8). In a recent study, hepatitis C virus was serially passaged more than 50 times with a small bottleneck (about 10-fold dilution), which resulted in a decrease in drug sensitivity along with an increase in genetic diversity (9). Understanding the transition of a viral population structure during host-to-host transmissions may help us determine the implications of the occurrence of virulent viral variants in the future.

In this study, we investigated a change in population structure during serial passages (bottleneck events) using rhesus rotavirus (RRV) as a model virus. Rotavirus, a double-stranded RNA (dsRNA) virus with 11 genome segments, exists as a quasispecies (10), is transmitted via the fecal-oral route, and causes severe diarrhea among children under 5 years old. Despite recommendations to vaccinate and improve hygiene (11), rotavirus outbreaks have occurred sporadically both in developed and developing countries (12–15). Assuming human-to-human transmission, RRV was serially passaged under weaker or stronger bottleneck events (multiplicity of infection [MOI] of 0.1 or 0.001, respectively) without any positive selections. During serial passages, changes to three phenotypes (infectious titer, cell binding ability, and specific growth rate) were confirmed. We then estimated the nucleotide diversity of 11 genome segments as an indicator of genetic diversity, and a rank abundance analysis was performed to confirm whether a mutant swarm was expanded or reduced using the single-nucleotide polymorphism (SNP) information. The synonymous (*dS*) and nonsynonymous (*dN*) substitution rates were also calculated to examine the exertion of any selective pressures on RRV genome segments. The evolutionary rate of 11 genome segments was estimated using the BEAST2 software package (16). Finally, we confirmed the effect of lower frequency mutants on phenotypic changes to a population by simulation using the estimated subpopulation frequency. Based on these results, the relationship between phenotypes and genetic diversity of an RRV population is discussed.

## RESULTS

### Infectious titer, cell binding ability, and specific growth rate.

**FIG 1:**
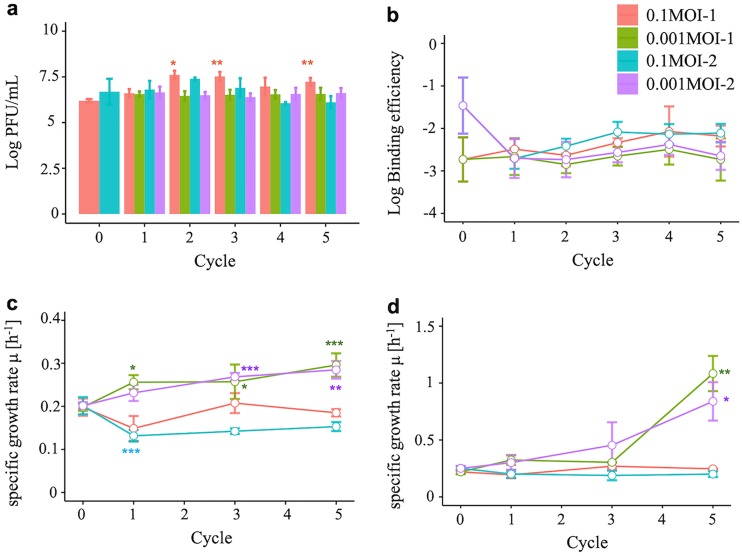
Infectious titer, cell binding ability, and specific growth rate. Numbers of PFU/ml as infectious titer (a), host cell binding efficiency (b), specific growth rate estimated from a one-step growth curve (c), and values from a modified Gompertz model (d) were estimated to confirm whether the serial passages altered the RRV phenotypes. All experiments were conducted three times. The error bar indicates the standard deviations. While no significant differences in cell binding ability were observed, there were significant differences in infectious titer between the original population and the 0.1MOI-1_2, 0.1MOI-1_3, and 0.1MOI-1_5 populations. The specific growth rates of 0.001MOI-1_5 and 0.001MOI-2_5 populations, which were derived from both the one-step growth curve and the modified Gompertz model, were also significantly larger than those of the original population (**, *P* < 0.01; *, *P* < 0.05; Student's *t* test).

RRV populations were serially passaged five times at different multiplicities of infection (MOI; 0.1 or 0.001). Infectious titers ranged from 10^6^ to 10^7.5^ PFU/ml, and only the 0.1MOI-1_2, 0.1MOI-1_3, and 0.1MOI-1_5 lineages showed significant increases in infectious titer from that of the original population (*P* values of 0.015, 0.006, and 0.007, respectively) (Fig. 1a). (We denote the RRV populations obtained from the 1st and 2nd serial passages at an MOI of 0.1 as 0.1MOI-1 and 0.1MOI-2 and those at an MOI of 0.001 as 0.001MOI-1 and 0.001MOI-2. The number following each population code name is the passage number, e.g., 0.1MOI-1_5 is the 1st population lineage passaged five times at an MOI of 0.1.) The cell binding ability was indicated by the binding efficiency to cells (the proportion of genome copies from virions bound to cells to those in the inoculum) (Fig. 1b). Fewer virions (less than 2%) were able to bind to cells, but no statistical changes were observed during serial passages. The specific growth rate was derived from the one-step growth curve by calculating the slope at the logarithmic phase and the modified Gompertz model by the curve approximating the time course data for infectious titers (Fig. 1c and d). Average (± standard deviation) specific growth rates, estimated from a one-step growth curve, of 0.001MOI lineages tended to be higher than those of 0.1MOI lineages (Fig. 1c). The 1st and 2nd 0.001MOI_5 populations showed especially significant differences from initial populations (*P* < 0.01). The values of specific growth rates of 0.001MOI_5 populations estimated by the modified Gompertz model were also significantly higher than those of initial populations (Fig. 1d; *P* < 0.05).

### Quality and average coverage of next-generation sequencing (NGS) samples.

Average coverage per site of 240 out of 242 samples was more than 250, which is a reference value recommended previously (17), but the others (NSP2 and NSP5/6 of 0.001MOI-1_4) showed average coverage of 189 and 188 (Table 1 and 2). We found high-quality scores for these low-coverage regions (32.9 on average, equal to less than a 0.1% sequence error), so we used these sequence data in the analysis. Nucleotide sequences from 626 to 637 in the VP1 genome segment of the 0.1MOI lineage were not used for analysis due to low coverage.

**TABLE 1:** Average coverage of each genome segment of the first lineage

Segment	Coverage for cycle:										
	0	1		2		3		4		5	
		0.1MOI	0.001MOI	0.1MOI	0.001MOI	0.1MOI	0.001MOI	0.1MOI	0.001MOI	0.1MOI	0.001MOI
VP1	644	667	5,791	1,425	5,365	412	1,473	267	534	681	3,859
VP2	952	877	6,352	1,334	5,792	633	2,181	356	729	1,014	4,898
VP3	2,872	2,457	5,608	3,915	5,184	1,660	1,667	1,174	624	2,344	4,432
VP4	1,310	1,273	6,190	2,495	5,972	781	1,804	628	719	1,424	4,477
VP6	2,070	2,271	7,272	4,636	6,464	1,288	1,618	1,399	432	3,256	5,242
VP7	2,748	3,192	6,888	5,944	6,087	2,034	1,409	2,314	412	4,660	5,224
NSP1	997	964	3,672	1,707	3,591	553	819	467	302	996	3,050
NSP2	2,242	2,561	2,692	5,610	3,672	1,482	712	1,787	189	3,026	2,528
NSP3	3,267	3,231	6,350	5,033	4,479	2,192	1,251	2,413	307	5,610	5,035
NSP4	3,267	3,231	6,350	5,033	4,479	2,192	1,251	2,413	307	5,610	5,035
NSP5/6	6,613	6,624	7,561	7,840	7,290	6,299	2,085	5,677	188	7,327	6,173

**TABLE 2:** Average coverage of each genome segment of the 2nd lineage

Segment	Coverage for cycle:										
	0	1		2		3		4		5	
		0.1MOI	0.001MOI	0.1MOI	0.001MOI	0.1MOI	0.001MOI	0.1MOI	0.001MOI	0.1MOI	0.001MOI
VP1	1,651	1,280	5,649	1,287	4,033	1,369	1,827	1,434	5,192	718	5,075
VP2	1,997	1,573	5,875	1,871	5,323	2,381	3,060	977	6,192	2,581	5,864
VP3	780	2,485	5,193	2,761	4,151	2,291	2,006	1,358	5,711	2,797	5,422
VP4	828	2,245	5,948	2,276	3,970	2,391	2,261	1,147	5,473	2,587	5,312
VP6	955	2,094	6,119	2,500	4,439	2,651	2,123	979	7,093	2,295	6,605
VP7	814	3,204	5,936	3,289	3,524	3,752	2,200	1,593	7,217	3,517	6,777
NSP1	605	1,149	3,469	1,887	2,820	1,983	1,114	703	4,379	1,929	4,380
NSP2	750	1,821	3,185	3,125	1,425	2,571	875	745	4,041	2,420	4,262
NSP3	571	1,548	4,541	2,413	4,251	2,228	1,754	721	7,337	2,497	5,886
NSP4	570	1,547	4,541	2,411	4,251	2,227	1,754	720	7,337	2,495	5,886
NSP5/6	1,000	5,137	7,446	6,024	4,409	5,629	3,760	1,616	7,707	5,399	7,186

### Nucleotide diversity of RRV populations.

**FIG 2:**
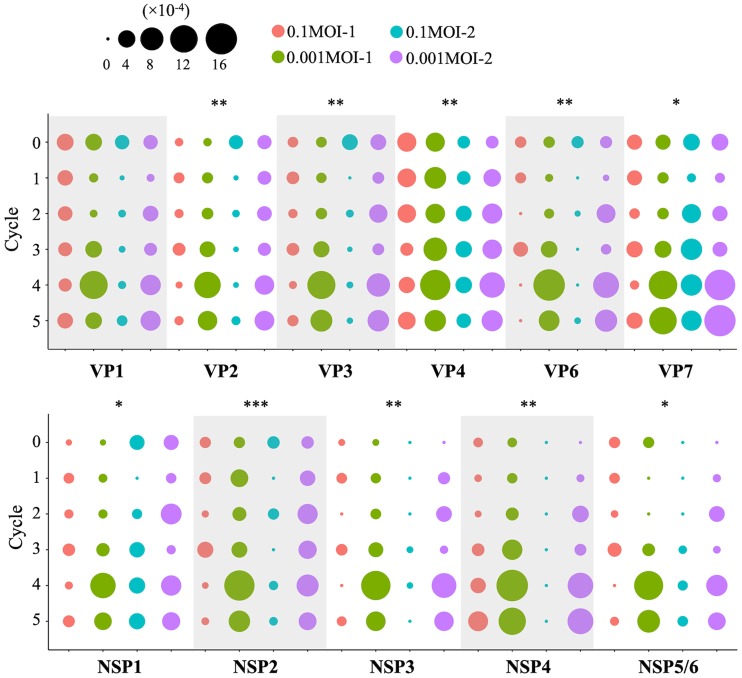
Genetic diversity of rhesus rotavirus populations. Transition of nucleotide diversity of all rhesus rotavirus (RRV) populations is displayed as a bubble chart. The size of the plot corresponds to the value of nucleotide diversity (from 0 to 1.6 × 10^−3^). The nucleotide diversity of RRV populations serially passaged under stronger bottlenecks (0.001MOI population) increased at later stages of passage, and ANOVA revealed the significant differences between 0.1MOI and 0.001MOI populations of each lineage except for the VP1 genome segment (*, *P* < 0.5; **, *P* < 0.01; ***, *P* < 0.001).

Using the frequency of SNPs, the nucleotide diversity of each genome segment was calculated as an indicator of the genetic diversity of an RRV population. Nucleotide diversity is defined as the proportion of nucleotide difference observed when two copies of the genome are sampled, and the transitions of nucleotide diversity through serial passages were expressed by the change of bubble plot size (Fig. 2). Among the 0.001MOI lineages, the increments of nucleotide diversity after cycle 4 were seen in VP1, VP2, VP3, VP6, VP7, NSP3, NSP4, and NSP5/6 genome segments. Significant differences in the effect on nucleotide diversity between an MOI of 0.1 and 0.001 were observed for all segments, except for the VP1 genome segment (*P* = 0.058), by using analysis of variance (ANOVA). This result indicated that RRV populations passaged repeatedly under a stronger bottleneck expanded the mutant swarm.

### Rank abundance distribution.

**FIG 3:**
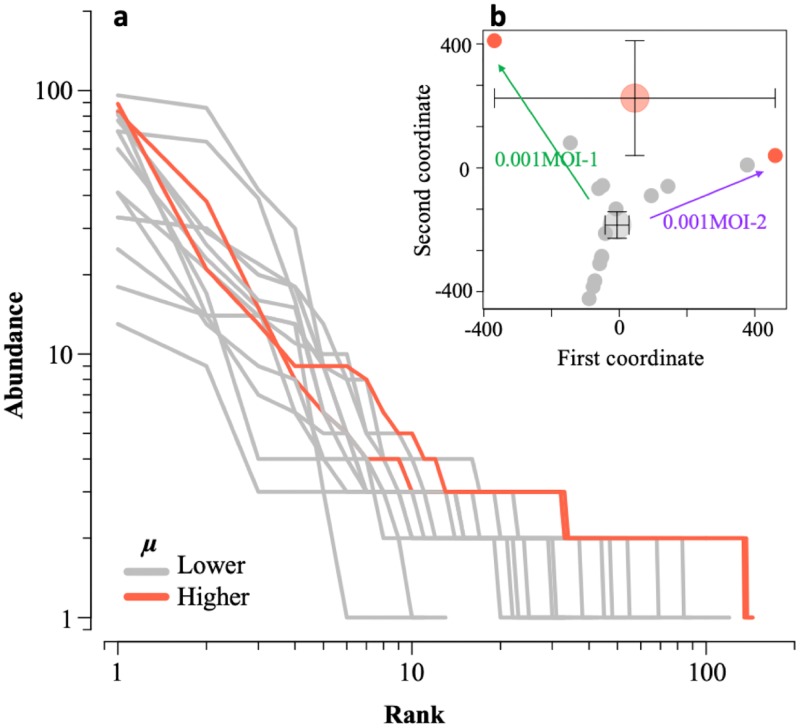
Expansion of minor mutant swarms and drastic deviations in populations showing higher genetic diversity. (a) Rank abundance curves of 0.1- and 0.001MOI_0, 0.001MOI_1, 0.001MOI_3, and 0.001MOI_5 populations were estimated based on SNP variety and frequency (gray, populations with lower specific growth rate, *μ*; red, two populations with higher *μ*). Two populations showing higher specific growth rate displayed similar shapes of stratification, because the abundance of low-rank (from ten to a hundred) SNPs remained around two or three percent in the population, which implied minor sequences acquired spaces that originally were dominated by major sequences. (b) Dissimilarity of each population structure was displayed as multidimensional scaling based on the SNP frequency. Small circles (orange, populations with higher *μ*; gray, other populations) indicate the relative locations of each population on two dimensions, as determined by SNP frequency. Large circles with error bars indicate the averaged locations of two classes based on the degree of *μ* in a two-dimensional area. Populations with smaller *μ* were not so different from each other, since the standard deviation of a large circle was small. On the other hand, larger standard deviations of populations with higher *μ* indicated that structures of two populations showing higher growth rate were greatly diverged despite the similar stratification of the mutant swarm, as shown in panel a.

Nucleotide diversity estimation in Fig. 2 implied the expansion of the mutant swarm, but it remained unclear whether the number of minor mutants increased or a new competitor for the master sequence appeared (both increased the population diversity). The elucidation of a layer structure in virus populations can help us gain insight into reasons for the increases in the nucleotide diversity of 0.001MOI lineages, so we conducted a rank abundance analysis, which visualized the abundance of master and minor sequences in a population with each rank and revealed how population structures changed during our experiment. According to the level of the specific growth rate, 0.1- and 0.001MOI_0, 0.001MOI_1, 0.001MOI_3, and 0.001MOI_5 populations were grouped into two classes (two 0.001MOI_5 populations and others). Abundance in the rank abundance curve corresponding to each SNP frequency decreased with an increase in rank, which was determined by the degree of dominance in a population (Fig. 3a). The mutant swarm is regarded as expanded when the rank abundance curves are extended to the right side of the *x* axis or the abundance of minor sequences increases. Since rank abundance curves were formed in a divergent shape from a rank of 0 to 100, all RRV populations included the mutant swarm for a number of minor sequences, which indicates that the RRV population exists as a quasispecies. The behavior of curves of 0.001MOI_5 populations became similar to each other, and both the rank and the abundance of minor sequences of 0.001MOI_5 populations increased more than those of other populations. These results indicated that minor mutant swarms of 0.001MOI lineages expanded during the serial passages.

Similar behavior of rank abundance curves in 0.001MOI_5 populations showing a higher specific growth rate was confirmed, but there was the possibility of the dissimilarity of population structure compositions due to differences in SNP composition. We employed multidimensional scaling to examine the dissimilarity of RRV populations based on the distance determined by SNP information for all populations. As a result, 0.001MOI_5 populations were distant from each other, and the *x* deviation of mean location of 0.001MOI_5 populations was larger than that of other populations (Fig. 3b). This indicates that population structure compositions of the populations are different from each other despite similar population structure shapes, as shown in Fig. 3a. Populations passaged in the stronger bottleneck tended to become diverged largely as virus passage continued, but the direction of evolutionary divergence could be different, which can be explained by genetic drift.

### The transition of SNP frequency.

**FIG 4:**
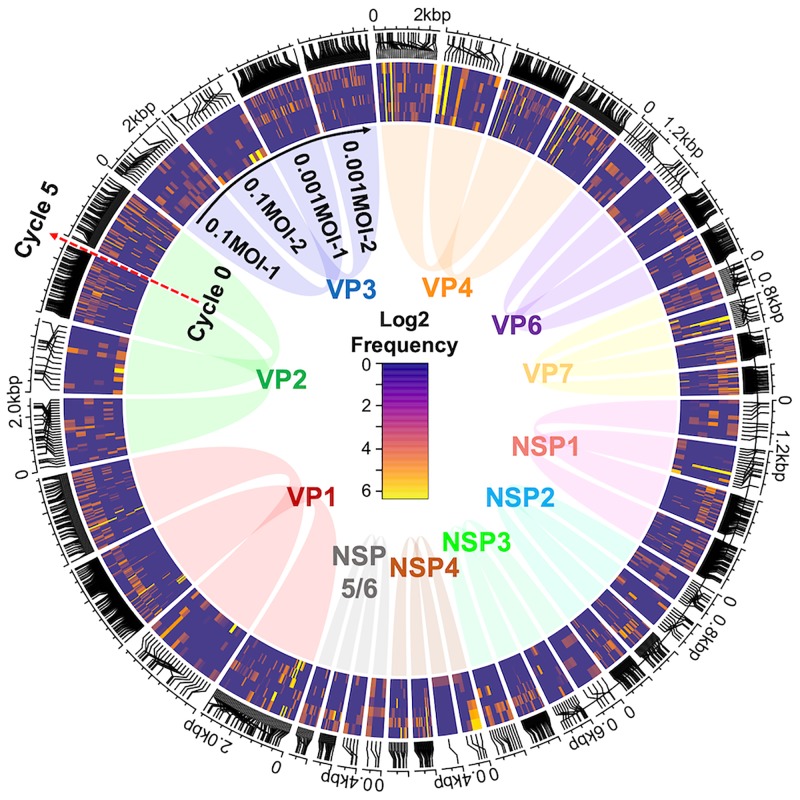
Circos plot of heatmaps of SNPs in four lineages and their frequencies. The frequency of each SNP was transformed to the base 2 logarithm, corresponding to color (high frequency, yellow; low frequency, navy), and the changes of SNP frequency during serial passages were seen from inside to outside the Circos plot (red arrow). A heatmap cell of four lineages was arranged clockwise from VP1 to NSP5/6, showing the locations of SNPs on genome segments. Lineages subjected to the stronger bottleneck effect (0.001MOI) increased the number of SNPs at the later stage of virus passage (brightly colored cells [orange or yellow] increased after cycle 3 or 4), and several SNPs randomly appeared and disappeared.

Rank abundance analysis revealed that the content of layer structures in populations of 0.001MOI lineages were distinct from each other despite similar shapes of the structures, indicating the different profiles of SNPs (type and/or frequency). SNPs in the entire genomic region and their frequency (converted by base 2 logarithm) were shown as a heatmap of a Circos plot, and the change of color in the heatmap expressed the transition of SNP frequency during serial passages (inside, initial; outside, cycle 5 populations) (Fig. 4). Unlike the 0.1MOI lineages, there were a number of heatmap cells of 0.001MOI lineages, indicating that relatively more SNPs appeared in 0.001MOI lineages. The frequency of several SNPs of 0.001MOI lineages increased after cycle 4. On the other hand, several SNPs suddenly appeared in or disappeared from a population. We also found dominant mutations in reference sequences (Table 3). Two replacements on consensus sequences were observed, VP4:V184A (common to all lineages) and VP7:E256G (specific to the 0.1MOI-2 lineage), which gradually increased and were finally fixed in the population.

**TABLE 3:** Amino acid difference between the reference and consensus sequences obtained in this study

Cycle	Amino acid difference by position^*a*^ (reference→substitution)														
	VP3:501 (G→R), common	VP4:36 (P→L), common	VP4:180 (K→E), common	VP4:184 (V→A)				VP4:187 (R→K), common	VP4:267 (C→Y), common	VP4:379 (T→I), common	VP6:245 (T→A), common	VP7:256 (E→G)			
				a	b	c	d					a	b	c	d
0	100	100	100	33.4	33.4	2.8	2.8	100	100	100	100	0	0	0	0
1	100	100	100	25.2	60.3	13.5	14.4	100	100	100	100	0	0	2.6	0
2	100	100	100	44.1	68.4	39.4	69.0	100	100	100	100	0	0	34.2	0
3	100	100	100	70.5	77.4	64.0	80.5	100	100	100	100	0	0	70.4	0
4	100	100	100	79.5	76.4	73.8	87.0	100	100	100	100	0	0	72.0	0
5	100	100	100	86.4	83.1	83.6	89.0	100	100	100	100	0	0	96.2	0

a Values show the frequency (%) of sequences possessing a substitution. a, 0.1MOI-1 lineage; b, 0.001MOI-1 lineage; c, 0.1MOI-2 lineage; d, 0.001MOI-2 lineage; common, common to all lineages.

### Nonsynonymous and synonymous substitutions.

**FIG 5:**
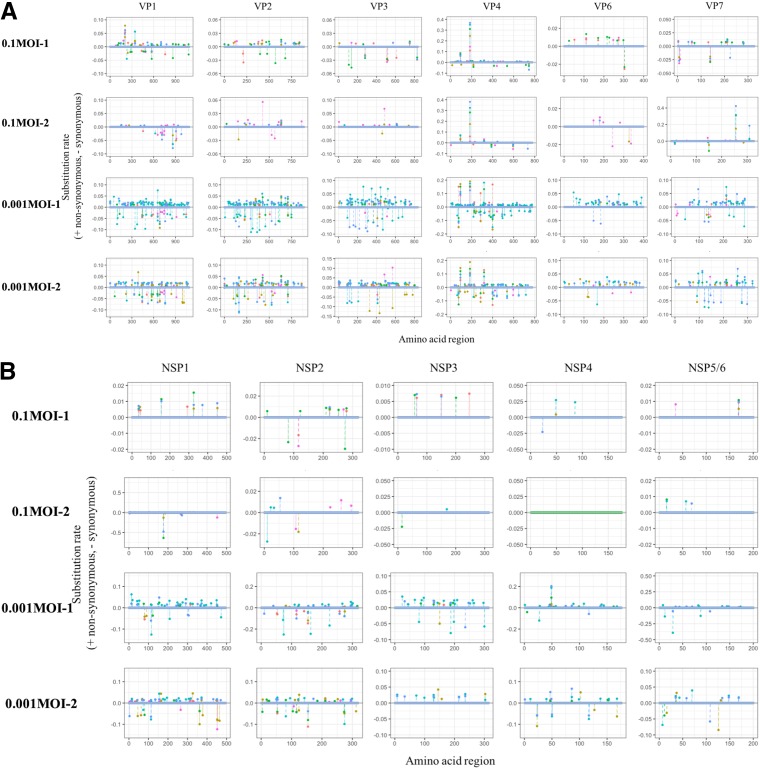
Location of the nonsynonymous and synonymous substitutions. The nonsynonymous substitution rate (*dN*; causing amino acid replacement) was expressed as a positive value, whereas the synonymous rate (*dS*; causing only nucleotide replacement) was shown as a negative value on each amino acid sequence (pink, initial; orange, cycle 1; dark yellow, cycle 2; green, cycle 3; cyan, cycle 4; blue, cycle 5). (A) Genome segments coding for viral proteins (VPs). (B) Genome segments coding for nonstructural proteins (NSPs). Both 0.1- and 0.001MOI lineages tended to include more nonsynonymous substitutions than synonymous substitutions. The substitutions of 0.001MOI lineages were distributed more densely among the genome segment than 0.1MOI lineages. In 0.001MOI linages the *dS* showed higher values, but nonsynonymous substitutions showed higher numbers than synonymous substitutions despite *dN* values being small.

Nonsynonymous substitution causes amino acid replacement, while synonymous substitution just changes the nucleotide sequence. Observed nonsynonymous and synonymous substitutions were plotted as positive and negative values, respectively, in the order of the 5′ to 3′ ends of each genome segment (Fig. 5). The number of nonsynonymous substitutions of the 0.001MOI lineages tends to be higher than that of 0.1MOI lineages. In the case of the VP4 genome segment, the *dN* values were comparable to the *dS* values. The substitutions of all genome segments occurred randomly and thoroughly, although the spike head (∼65- to 224-amino-acid sequence) and antigen domain (∼250- to 480-amino-acid sequence) regions of the VP4 genome segment showed high variability.

### Transition of the difference between *dN* and *dS*.

**FIG 6:**
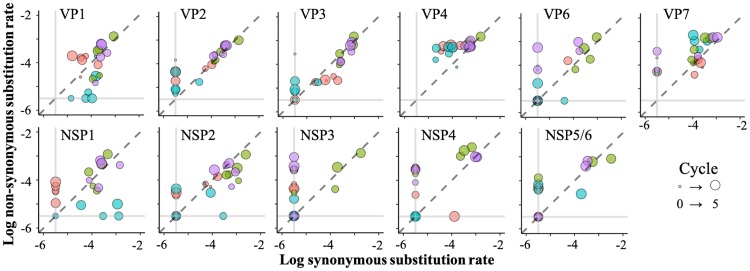
Overall proportion of nonsynonymous and synonymous substitutions in each genome segment. The proportion of logarithmic nonsynonymous (*dN*; causing amino acid replacement) to synonymous (*dS*; causing only nucleotide replacement) substitution rate is displayed, and the size of circles corresponds to the passage numbers (red, 0.1MOI-1; green, 0.001MOI-1; blue, 0.1MOI-2; pink, 0.001MOI-2). The dashed line indicates that the proportion of *dN* and *dS* is balanced (the population structure is explained by a random change of SNP frequency), and plots on two vertical gray lines mean that the value of *dN* or *dS* is zero. Plots of 0.001MOI lineages were gathered around dashed lines, which meant that *dN* and *dS* were equal and implied genetic neutrality. On the other hand, plots of 0.1MOI populations tended to disperse from dashed lines.

The difference between rates of nonsynonymous (*dN*) and synonymous (*dS*) substitution is used as an indicator of the existence of natural selection at a certain region of the genome (*dN* > *dS*, positive selection; *dS* > *dN*, negative selection; *dN* = *dS*, genetic neutrality, in which the gene is neither advantageous nor disadvantageous for survival). In Fig. 6, logarithmic *dS* and *dN* of each genome segment were compared, and plots above the *dN* = *dS* line indicate the existence of positive selection, while negative selection can be found when plots were below the *dN* = *dS* line. Plots on the *dN* = *dS* line indicate that the genome segment is affected by genetic drift. The *dN* and *dS* of the VP1 genome segment of the 0.001MOI lineages remained the same, although plots of the 0.1MOI lineages became slightly dispersed from the *dN* = *dS* line. In NSPs, genome segments in 0.1MOI lineage plots also tended to be dispersed from the *dN* = *dS* line, and the *dN* of NSPs tended to be zero. The *dN* and *dS* of VP2, VP3, VP4, VP7, NSP1, NSP2, NSP4 (at cycle 5), and NSP5/6 genome segments of the 0.001MOI lineages showed no difference, but the values of both substitution rates increased during serial passages. These results indicated that genetic drift was the main driver for the population structure change in 0.001MOI lineages.

### Evolutionary rate of 11 genome segments per cycle.

Evolutionary rate was calculated for every genome segment using BEAST2 software (Table 4). The NSP4 evolutionary rate of the 0.1MOI-2 lineage could not be calculated because no SNPs were detected. Shorter genome segments, such as NSP4 (750 bp) and NSP5/6 (667 bp), displayed a relatively high evolutionary rate (about 10^−4^ substitutions/site/cycle). On the other hand, VP1 (3,267 bp), VP2 (2,664 bp), VP3 (2,508 bp), and VP4 (2,362 bp) genome segments showed ∼10^−5^ substitutions/site/cycle. Middle-sized genome segments (VP6, VP7, NSP1, NSP2, and NSP3) were different between the MOI settings. The evolutionary rate of all genome segments of 0.1MOI lineages were higher than those of 0.001MOI lineages.

**TABLE 4:** Evolutionary rate for rotavirus genome segments at nucleotide level

Lineage	Evolutionary rate^*a*^ (95% HPD^*b*^)										
	VP1 (3,267 bp)	VP2 (2,664 bp)	VP3 (2,508 bp)	VP4 (2,362 bp)	VP6 (1,356 bp)	VP7 (1,062 bp)	NSP1 (1,599 bp)	NSP2 (1,059 bp)	NSP3 (1,078 bp)	NSP4 (750 bp)	NSP5 (667 bp)
0.1MOI-1	2.45 (0.05–5.69)	5.47 (0.09–11.9)	7.08 (0.01–17.0)	4.55 (0.06–10.9)	19.2 (3.76–44.8)	16.5 (0.04–41.2)	14.7 (0.01–36.7)	25.3 (0.07–55.7)	58.4 (2.22–38.9)	44.4 (1.91–122)	23.0 (0.20–61.2)
0.1MOI-2	3.60 (0.01–8.38)	5.32 (0.01–13.3)	6.66 (0.02–16.5)	5.10 (0.01–19.4)	57.2 (0.91–67.1)	12.8 (0.01–32.1)	11.0 (0.02–32.1)	19.7 (0.08–46.9)	29.6 (0.34–82.5)		4.93 (0.01–15.5)
0.001MOI-1	1.56 (0.02–13.0)	1.32 (0.03–5.30)	0.70 (0.01–7.56)	2.46 (0.04–39.5)	3.48 (0.02–20.3)	6.80 (0.05–15.8)	6.28 (0.02–30.2)	4.49 (1.14–14.0)	12.0 (0.89–28.9)	22.0 (3.38–46.3)	4.93 (0.01–15.5)
0.001MOI-2	1.68 (0.07–4.20)	1.94 (0.03–5.16)	2.20 (0.03–5.72)	1.69 (0.04–3.95)	5.95 (0.03–14.5)	8.56 (0.01–18.8)	7.29 (0.02–28.9)	6.67 (0.01–19.0)	18.9 (0.04–42.7)	25.6 (0.01–56.0)	21.2 (0.03–53.1)

a With 10^−5^ substitutions/nucleotide site/cycle. Genome lengths are shown as base pairs.

b HPD, highest posterior density.

### Simulation of effect of minor mutants on the specific growth rate of a population.

**FIG 7:**
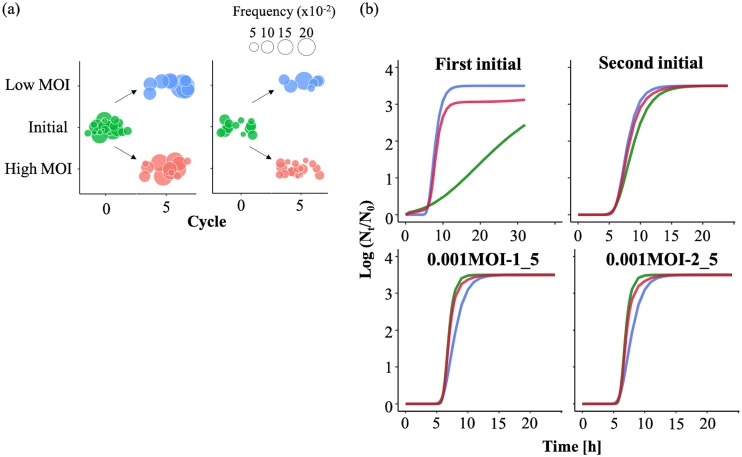
Size of subpopulations in a population and simulated growth curves. (a) The estimated composition of a population was illustrated based on the subpopulation (haplotype) frequency and the strength of the bottleneck effect. The size of a circle shows the subpopulation frequency (green, initial population; red, 0.1MOI lineages; blue, 0.001MOI lineages). Smaller subpopulations (less than 5%) remained in populations passaged at higher MOI, but lower MOI lineages were composed of several larger subpopulations. This implied that the stronger bottleneck (lower MOI) helped minor subpopulations gain sequence spaces and increase their frequency. (b) Three types of growth curves of rhesus rotavirus were shown separately, assuming an interaction among minor subpopulations (red, total population; blue, major population; green, an ensemble of minor subpopulations). It was hypothesized that subpopulations of less than 5% frequency decreased the diversity of a population, because it was probable that such a population included deleterious mutations, which wasted several sequence spaces. When the frequency of the subpopulation was more than 5%, the specific growth rate of a population increased, since higher frequency in a population implied some adaptivity (although it was weak or temporal).

The quasispecies structure of RRV was estimated based on haplotype analysis to confirm the effect of low-frequency subpopulations on the specific growth rate as a population by haplotype frequency, which was regarded as the proportion of the subpopulation including minor mutants. The number of subpopulations and its frequency in initial and cycle 5 populations are displayed in Fig. 7a. Low-frequency subpopulations were included in both initial populations and remained in populations passaged under the weaker bottleneck, while larger sizes of subpopulations passaged under stronger bottlenecks were found (Fig. 7a). Because this estimation implied the expansion of the mutant swarm, it is consistent with results of nucleotide diversity and rank abundance analyses (Fig. 2 and 3). Using the estimated subpopulation frequency of 0.001MOI populations, the specific growth rate of an entire population was simulated under three scenarios. In scenario 1, we assumed that specific growth rates correlated with the frequency of each population classified into a major population and some subpopulations (less than 10% frequency). The specific growth rate of a population was recalculated by summing each populations’ growth curve. The specific growth rates of the populations passaged five times under stronger bottlenecks were almost the same as those of initial populations (Table 5). We then changed the criterion of subpopulation frequency (less than 20% frequency) in scenario 2. The specific growth rate of the 0.001MOI-1_5 population was doubled, but small changes in 0.001MOI-2_5 still were seen (Table 5). On the other hand, by assuming the existence of some interactions among subpopulations (scenario 3), the simulated specific growth rates of both 0.001MOI lineages diverged from those of initial populations, like the values observed in our experiments (Table 5 and Fig. 7b).

**TABLE 5:** Simulated specific growth rate of three scenarios

Simulation	μ_5_/μ_0_^*d*^	
	First lineage	Second lineage
1^*a*^	1.20	0.86
2^*b*^	1.95	1.15
3^*c*^	2.57	1.91
Observed	4.93	3.35

a Populations were defined as minor populations when frequency in a population was less than 10%. Population frequency positively correlated with specific growth rate for minor subpopulations.

b A major population was found at 80% frequency. Positive correlation between frequency and specific growth rate was assumed, similar to simulation 1.

c Populations of less than 5% decreased the specific growth rate of a minor population due to competition for survival, while populations of more than 5% increased the rate because of relatively higher frequency.

d The ratio of specific growth rate for populations passed five times under a stronger bottleneck than that of the initial population.

## DISCUSSION

In this study, RRV populations were serially passaged under stronger/weaker bottleneck conditions. The specific growth rate and the nucleotide diversity of 0.001MOI lineages (passaged at a lower MOI) increased during the latter part of serial passages (Fig. 1c and 2). The mutant swarms of two 0.001MOI lineages were expanded, as shown in Fig. 3, and a number of SNPs randomly appeared in or disappeared from RRV populations (Fig. 4). Almost all mutations of 0.001MOI lineages were selectively neutral, since synonymous and nonsynonymous substitutions were non-site-specifically located on genome segments (Fig. 4 and 5), and the values of *dN* and *dS* of 0.001MOI lineages were close to each other (Fig. 6). The evolutionary rates of each genome segment were estimated based on the SNP information (Table 4), and the values of 0.001MOI lineages were relatively lower. Simple simulations based on the modified Gompertz model showed that minor mutants could affect the phenotype as a population in a mutant frequency-dependent manner, with some interactions between minor mutants (Fig. 7).

A common amino acid replacement (VP4:V184A) was present in both initial populations at lower frequency and then became dominant among all lineages during serial passages (Table 3). This can be explained by the positive selection for some phenotypic advantages during the repeated cultivation of RRV, because population size was sporadically large enough for positive selection to act efficiently (18). The *dN* values of V184A being higher than *dS* values (Fig. 5 and 6) also supports that the V184A frequency in all lineages increase because of the positive selection. It seems that V184A is not beneficial to cell binding ability and specific growth rate (Fig. 1b, c, and d), because the V184A replacement is also observed in the 0.1MOI lineages (Table 3). However, further studies are needed to characterize other phenotypes, such as structural stability and cell tropism.

A bottleneck effect randomly eliminates genome sequences from a population (19) and promotes the evolution of the virus due to a drastic change of population structure. In this study, the nucleotide diversity of rotaviruses passaged under the stronger bottleneck effect increased more than that under the weaker bottleneck effect (Fig. 2). Lequime et al. reported that the genetic diversity of dengue virus during intrahost transmission was immediately replenished following a bottleneck event, although its expansion of a mutant swarm was also restricted by negative selection (6). Morelli et al. reported that a lot of minor sequences were transmitted during interhost transmission, where a bottleneck effect worked (7). Taking these findings together with our study, although a bottleneck effect temporarily decreases the population size, it can excavate minor sequences from a mutant swarm. The size of a mutant swarm then can be expanded to a level determined by the degree of heterogeneity of the population.

The concept of sequence space (20) provides more detailed information to address the question of why the increase in genetic diversity occurred in the lineages of viruses placed under the stronger bottleneck conditions. We assume the existence of a hidden layer of a mutant swarm, which includes minor mutations that cannot be distinguished from a sequencing error even in the next-generation sequencing. In this hidden-layer assumption, a mutant swarm is shaped into a layer structure, in which the layers of the detectable minor sequences (more than 1% frequency) and major sequences lie on a hidden layer composed of undetectable minor sequences. When a virus population undergoes a bottleneck, several minor sequences in the hidden layer of a mutant swarm accidentally appear by moving into a new sequence space. In the concept-of-fitness landscapes (i.e., higher fitness strains are on the top of the hill and lower fitness strains gather around the bottom of the hill) (5), the top of the hill is truncated by a bottleneck effect and then the substratum is expanded. In other words, the hill becomes less steep because the minor sequences originally existing in the hidden layer of a mutant swarm acquire some of the spaces of the upper layer in the fitness hill. Lauring and Andino suggested that mutants tend to gather in the upper part of the fitness hill if they show a low mutation rate (21), which is consistent with our results for evolutionary rates (Table 4). A bottleneck effect gives minor sequences a chance to explore a sequence space.

An expansion of mutant swarms appears to lead to an increase in the specific growth rate without minor sequences being dominant in the population. Serial passages are known to change viral phenotypes. For example, the rabies virus adapted to a new environment without the replacement of the master sequence during serial passages (4). The infectivity of simian/human immunodeficiency virus increased after serial passages on macaques as the size of the mutant swarm expanded (22). The replicative fitness of hepatitis C virus increased by serial passages under a bottleneck according to an expansion of the mutant swarm (9). It was also reported that the size of the mutant swarm positively correlated with the growth ability of the West Nile virus (3). These previous reports indicate that a larger mutant swarm can provide a virus population with the chance to acquire some advantageous phenotypes, which is also the case in the present study, in which the specific growth rate (relating to growth ability) increased under a bottleneck effect (Fig. 1c), as the size of the mutant swarm of the 0.001MOI lineages expanded (Fig. 3). We think that some minor sequences find new sequence spaces created by a bottleneck, resulting in an increase in the specific population growth rate. Fitness is an overall indicator of the ability to produce infectious progeny, such as binding efficiency to receptors, specific growth rate, and burst size (20). The fitness of mutants displaying a higher specific growth rate was lower in this study, since nucleotide replacements specific to 0.001MOI lineages on master sequences were not found (Table 3). If the fitness of a mutant is outstanding, the frequency of the mutant sequence in a population increases through passages and finally becomes dominant. Since many mutations were regarded as neutral in this study (Fig. 5) despite an increase in specific growth rate, we speculate that several minor sequences showed inferior aspects of fitness compared to those of the master sequences, compensating for higher growth ability.

Quasispecies reconstruction implied that the stronger bottleneck expanded the size of subpopulations (lineages including minor mutants) while smaller populations still remained in 0.1MOI lineages (Fig. 7a), which supports the hypothesis that several minor sequences acquire sequence space created by the stronger bottleneck. By simulation using results of quasispecies reconstruction, the specific growth rate, as a population phenotype, increased under the assumption of some interactions between subpopulations and in a frequency-dependent manner (Fig. 7b). Interactions such as competition and cooperation between mutants have been already reported and can affect the viral population phenotype (23, 24). The supposed interaction in this study, for example, is that virions invade host cells by aggregation resulting from various electrostatic potentials of each mutant, which is supported by the existence of many nonsynonymous substitutions that can change the electrostatic potential of the virions (Fig. 5).

Rotavirus outbreaks are still being reported even after the implementation of vaccine programs. Some cases of rotavirus in California were reported despite a vaccination program and hygienic interventions, such as hand washing and disinfection (14). Zeller et al. found that vaccination did not affect rotavirus population size, although some unique clusters were found after vaccination (25). Therefore, rotavirus persistence in human society should be explained not only by the introduction of a vaccine but also by other factors. A bottleneck effect often can be seen in the life cycle of rotaviruses in the real environment (20). Human-to-human transmission corresponds to this situation of a bottleneck effect, since a few virions from feces attached to hands can be transmitted to new, susceptible humans. Interventions such as those in California may cause a bottleneck effect for rotavirus. Although information related to the size of mutant swarms of rotavirus populations is rarely obtained from outbreak cases, to understand the epidemic pattern of rotaviruses, we need to explore the transition of the genetic population structure of an outbreak-associated strain with molecular epidemiological studies using next-generation sequencing.

## MATERIALS AND METHODS

### Virus and cells.

MA104 cell lines (CRL-2378.1; ATCC) were grown in Eagle’s minimal essential medium (MEM) containing 10% fetal bovine serum (FBS), 2 mM l-glutamine, 1% penicillin-streptomycin (GIBCO by Life Technology), and 1.125 g/liter sodium bicarbonate (Wako Pure Chemical Industries, Ltd.) in a T75 flask. Average cell numbers were approximately 5.0 × 10^6^ cells/T75 flask in this study. We possessed two lineages of RRV (genotype G3P[3]) derived from the same ancestral population, and two descendant populations were used for serial passages, called initial populations.

### Serial passages.

The RRV suspension (10^6^ to 10^7^ PFU/ml) was diluted with serum-free MEM to adjust the MOI to 0.1 (5.0 × 10^5^ PFU/ml) or 0.001 (5.0 × 10^3^ PFU/ml), and 4 μl of 1 μg/μl trypsin from porcine pancreas was added. This mixed suspension, in a 1.5-ml tube, was put in an incubator at 37°C with 5% CO_2_ for 30 min. After incubation, the medium in a T75 flask was removed and washed twice with Dulbecco’s phosphate-buffered saline (−) [PBS(−); does not include Ca2^+^ and Mg2^+^, while PBS(+) includes both cations] (Nissui Pharmaceutical Co., Ltd.), and then 1 ml of RRV suspension was inoculated onto the confluent MA104 cells. The flask was incubated at 37°C with 5% CO_2_ for 60 min. After incubation, 32 ml of serum-free Eagle’s MEM was added to the MA104 cells, and the cells were reincubated at 37°C with 5% CO_2_ for 2 or 3 days. The freeze-melt cycle was done three times after incubation. The suspension that had been moved into a 50-ml tube from the flask was centrifuged at 12,600 × *g* for 10 min at 4°C and filtered with a 0.2-μm filter to remove the cell fractions. The collected RRV suspension was inoculated into the new MA104 cell again. The RRV population was passaged five times (cycles 0, 1, 2, 3, 4, and 5), and this series of experiments was conducted by using two different initial populations (the 1st and 2nd lineages). We denote the RRV populations obtained from the 1st and 2nd serial passages at an MOI of 0.1 as 0.1MOI-1 and 0.1MOI-2 and those at an MOI of 0.001 as 0.001MOI-1 and 0.001MOI-2. The number following each population code name is the passage number, e.g., 0.1MOI-1_5 is the 1st population lineage passaged five times at an MOI of 0.1.

The infectious titer (PFU/ml) of each passage was measured in triplicate by plaque assay. According to our previous report (26), serially diluted RRV suspensions were treated with 1 μg/μl trypsin from a porcine pancreas. The RRV suspensions then were inoculated onto confluent MA104 cells in the 6-well plate, and the plate was incubated at 37°C with 5% CO_2_ for 90 min. After incubation, the virus inoculate was removed, and the cells were washed twice with Dulbecco’s PBS(−) and then overlaid with 2.5% agar, including 2% FBS, 2% penicillin-streptomycin, 4 mM l-glutamine, 2.25 g/liter NaHCO_3_, and 4 μg/ml trypsin from porcine pancreas. The 6-well plates were incubated for 2 days and then dyed with 0.015% neutral red for 3 h. After 1 or 2 days, the plaque numbers were counted.

### Cell binding assay.

A cell binding assay was conducted in triplicate according to the steps outlined in previous reports (26, 27). MA104 cells were incubated on the 24-well plate for 2 or 3 days, and then the cell culture medium was removed and washed twice with Tris-buffered saline (TBS). The 100-μl samples of suspensions containing RRV (MOI of 1.0) were inoculated into each well and incubated at 4°C for 1 h with agitation every 15 min. After incubating and removing the virus suspension, the cells were washed three times with TBS, and then 140 μl of Dulbecco’s PBS and 560 μl of RNA extraction buffer (buffer AVL from the QIAamp viral RNA minikit; Qiagen, Germany) were added to each well of the plate. According to the manufacturer’s protocol, dsRNA then was purified and recovered. The extracted dsRNA was denatured at 95°C for 5 min and placed on ice for 2 min. The denatured dsRNA (single-stranded RNA) was reverse transcribed to cDNA using the PrimeScript RT reagent kit (perfect real time) (TaKaRa Bio, Inc., Kusatsu, Japan) according to the manufacturer’s protocol. The amount of cDNA was quantified by quantitative PCR (qPCR). The qPCR was performed with Premix *Ex Taq* (perfect real time) and a TaqMan probe in an Applied Biosystems 7500 real-time PCR system. The sequences of forward and reverse primers targeted to the NSP3 region were suggested by Pang et al. (forward, 5′-ACCATCTACACATGACCCTC-3′; reverse, 5′-GGTCACATAACGCCCC-3′) (28). The sequence of the TaqMan probe suggested by Pang et al. was modified slightly (5′-5-6-carboxyfluorescein-ATGAGCACA-ZEN-ATAGTTAAAAGCTAACACTGTCAA-3′). The qPCR amplification was performed with 1 cycle of an initial denaturation step at 95°C for 5 min, followed by 45 cycles of 94°C for 20 s and 60°C for 1 min. Finally, 1 cycle of 72°C for 5 min was conducted for extension. Genome copies (*G_t_*) of virus particles attached to cell surfaces were compared to those inoculated into cells (*G_0_*), and then binding efficiency was calculated as log(*G_t_/G_0_*).

### Specific growth rate.

RRV growth curves of each population were confirmed to obtain the specific growth rate. By following the recommendations from our previous report (26), MA104 cells were inoculated on the T75 flask for 2 or 3 days, and then the cell culture medium was removed and washed twice with Dulbecco’s PBS. The 400-μl samples of suspensions containing RRV (MOI of 0.01) were inoculated into the flasks individually. The supernatants were taken after 0, 6, 12, 18, 24, and 36 h, and then the infectious titer was measured by the plaque assay as described above. The series of this experiment was replicated three times (*n* = 3).

The specific growth rate was estimated in two ways by calculating the slope of one-step growth curves and by applying the modified Gompertz model. The one-step growth curve has been used to check virus growth by connecting observed log(PFU/ml) of virus at each sampling point [log(*N_t_*)], and the slope at the logarithmic phase (from 6 to 24 h in this study) shows the virus growth rate, *μ* (29). The modified Gompertz model, originally developed for bacterial growth (30), also has been used for viral capsid assembly and insect virus growth (31, 32), and it gives us three quantitative parameters (specific growth rate, the time at which viral progeny appears, and number of PFU/ml to reach the maximum value) of microbial growth. The modified Gompertz model showed a satisfactory goodness of fit of the poliovirus growth curve (33), so we estimated the specific growth rate using the modified Gompertz model in addition to the one-step growth curve. For estimating the specific growth rate, *μ*, at logarithmic growth phase, the modified Gompertz model was applied to the data set (30):

$$\text{log(}N_{t})=\text{log(}N_{0})+A\cdot \text{exp\{}-\text{exp[}1+\mu e\text{(}\lambda -t\text{)}/A\text{]\}}$$

where log(*N_0_*) is the logarithmic value of virus infectious titer (PFU/ml) before inoculation, *A* is the asymptotic value [log(*N_∞_*/*N_0_*)], *μ* is the specific growth rate (per hour), *e* is the Napier’s constant, and *λ* is the lag period (hours). These parameters (*μ*, *λ*, and *A*) were determined by the least-squares method, which minimized the distance between observed and simulated log(*N_t_*/*N_0_*) at each sampling point.

### FLDS.

Fragmented and primer-ligated dsRNA sequencing (FLDS), a method of cDNA library construction and sequencing for dsRNA, was utilized to obtain higher sequence coverage of RRV genome sequences than that of conventional transcriptome sequencing (RNA-seq) (34, 35). Briefly, the total nucleic acid in each sample of serial passages was extracted by phenol-chloroform (pH 5.2), and the extracted dsRNA was purified by cellulose D (Advantec, Tokyo, Japan). The dsRNA was fragmented physically by Covaris S220 (Woburn, MA, USA) and then reverse transcribed by a SMARTer rapid amplification of cDNA ends 5′/3′ kit (TaKaRa Bio, Inc.). The cDNA was amplified using KOD-Plus neo containing high-fidelity polymerase (Toyobo Co. Ltd., Japan). Small-sized cDNAs and primer dimers were removed by SPRI select (Beckman Coulter, Inc.). Fragmentation by Covaris S220 was conducted again to make the 400-bp cDNA, which was then ligated with an adapter (KAPA Biosystems, Woburn, MA, USA) for Illumina MiSeq. The concentration of the cDNA library was quantified by a KAPA library quantification kit (KAPA Biosystems). Each 300-bp fragment of the paired-end sequences was determined by Illumina MiSeq (Illumina, Inc., San Diego, CA, USA).

MiSeq sequencing reads underwent adaptor clipping and quality trimming using Trimmomatic v0.33 (36). PhiX sequences derived from control libraries and experimentally contaminated sequences were also removed using a mapping tool, Bowtie2 ver. 2.2.9 (37). Primer sequences used for cDNA synthesis and amplification were trimmed with Cutadapt ver. 1.15 (38). Low-complexity reads and PCR duplicates were detected and removed with PRINSEQ ver. 0.20.4 (39). The processed reads were used for mapping to the reference genome and generating the consensus sequence by CLC Genomics Workbench ver. 11 (CLC Bio, Aarhus, Denmark). Reference sequences of each RRV genome segment were obtained from NCBI using the following parameters: mismatch cost of 2, insertion/deletion cost of 3, length fraction of 0.8, and similarity fraction of 0.8. Using the function “Extract consensus sequences,” the consensus sequence of each genome segment was extracted from initial populations to utilize it as a reference sequence. Sequence reads in each population were mapped to the reference sequence (“Map reads to reference”) and then realigned locally to modify the gap generated in mapping to the reference sequences (“Local realignments”). Sequence coverages of RRV genome segments were obtained by applying mapped and locally realigned read files to SAMtools ver. 1.8 (40).

### Calling single-nucleotide polymorphisms.

The frequency of single-nucleotide polymorphisms (SNPs), which are mutations fixed in a population after undergoing selection or a bottleneck effect, was calculated by “Low frequency variant detection,” which is a function of CLC Genomics Workbench and detects SNPs based on a Bayesian model (both prior and posterior probability are estimated by an expectation-maximization algorithm, which is one of the maximum likelihood estimations). The Bayesian model employing the “Low frequency variant detection” function can statistically differentiate true SNPs from sequencing errors. The error rate threshold value was set to 1.0% to avoid false-positive variant detection according to previous studies (41, 42). SNPs showing fewer than ten sequence coverages also were filtered out by following the default setting of CLC Genomics Workbench. To confirm whether the observed SNPs caused amino acid substitutions, the “Amino acid changes” function of CLC Genomics Workbench was used. All SNPs on each genome segment and its frequency were displayed using “circlize” of the R software package (43).

### Estimation of nucleotide diversity and synonymous and nonsynonymous substitution rates.

The software SNPGenie was used to calculate the nucleotide diversity and nonsynonymous (*dN*) and synonymous (*dS*) substitution rates (44, 45). Nucleotide diversity is an indicator of genetic diversity in a population and indicates the probability that differences at the site on the genome are found between two sequences randomly sampled from a population. Nucleotide diversity is expressed as (46)

$$\pi \text{ = }\sum_{i\text{=1}}^{s}D_{i}\text{/}L$$

where *s* is the site-finding SNPs, *D_i_* is the proportion of nucleotide differences at the *i*th site, and *L* is the genome segment length. Synonymous (causing not amino acid but nucleotide replacement) and nonsynonymous (causing amino acid replacement) substitution rates were calculated in a manner similar to that for nucleotide diversity, but *D_i_* was modified according to *dS* or *dN* pairs (46). By comparing *dN* and *dS*, the existence of natural selection can be confirmed under the hypothesis that a nonsynonymous substitution can cause both adaptive and deleterious changes in protein composition on a virion.

### Rank abundance analysis.

Rank abundance distribution is one of the methods used to analyze the population structure by ordering individuals according to each level of dominance in a population (47). To compare and visualize the mutant distribution of 0.1- and 0.001MOI_0, 0.001MOI_1, 0.001MOI_3, and 0.001MOI_5 populations, abundance was assumed to be explained by SNP frequency and variety in this study, and then the SNPs were arranged according to rank based on frequency using the R software package RADanalysis (https://CRAN.R-project.org/package=RADanalysis). In addition, how each population structure differed was confirmed using multidimensional scaling, where distance between a pair of populations was calculated based on Manhattan distance, which was the sum of difference between two coordinates on the two-dimensional Euclidean space (e.g., the coordinates of each RRV population determined by their SNPs and its frequency).

### Evolutionary rate.

Evolutionary rate is the fixation rate of a mutation surviving from natural selection or bottleneck effect in a certain time period. By applying alignment files containing SNP sequences aligned by MEGA7 (48), the evolutionary rates of 11 genome segments were inferred, using BEAST2 software (http://www.beast2.org/), based on the Bayesian Markov chain Monte Carlo (MCMC) method (16). A general time-reversible (GTR) + γ model (a substitution model in which the rate of nucleotide replacement, *X*→*Y*, is identical with that of *Y*→*X*, and rates of nucleotide replacement are different in each genomic site) with a coalescent Bayesian skyline plot (a method for estimating past population dynamics changed by external factors, such as a bottleneck) was used to run the BEAST software, and then the output files were analyzed using TRACER software to extract the evolutionary rate of each genome segment (49).

### Simulation of an effect of minor mutants on the specific growth rate of a population.

We artificially reconstructed the quasispecies by TenSQR software (50), which enabled us to reconstruct quasispecies accurately even though quasispecies were composed of rare strains, and then we simulated the specific growth rate in the modified Gompertz model using the estimated haplotype (subpopulation) frequency under a variety of conditions, called simulations. In simulation 1, the populations with less than 10% frequency were defined as minor subpopulations, and the frequency was positively correlated with the specific growth rate of minor subpopulations. The specific growth rate of minor subpopulations, from 1% to 10% frequency, corresponded to 0.2 to 1.0 h^−1^. In simulation 2, a major population was regarded as a population of 80% frequency, and the residual population (20%) was regarded as a minor subpopulation. Positive correlation between frequency and specific growth rate was assumed as for simulation 1. In simulation 3, we assumed some interactions between minor populations of less than 10% frequency. Populations of less than 5% decreased the specific growth rate of a minor population due to competition for survival, while populations of more than 5% increased the rate because of relatively higher frequency, which possibly showed the capacity for adaptation (μ_sub_ = μ + Δμ_high_ − Δμ_low_, where μ_sub_ was the specific growth rate of a total minor population and *Δ*μ_high_ and Δμ_low_ were the amounts of change for populations of higher and lower frequency, respectively). The sum of the population size of a major population and minor mutants was regarded as the total population size [log(*N_t_*)]. The transition of total population sizes under each scenario was used to estimate the specific growth rate of a total population based on the modified Gompertz model under the assumption that the lag period (*λ* = 6.0 h) and the asymptote (*A *= 3.5 [log(*N_∞_/N_0_*)]) were identical for all strains.

### Statistical analysis.

A Student's *t* test was performed for infectious titer, cell binding ability, and specific growth rate between the original and the cycle 5 populations. ANOVA was conducted to confirm whether a stronger bottleneck affected nucleotide diversity. All statistical tests were performed using R software 3.5.0 (https://www.r-project.org/).

### Data availability.

Rotavirus sequence data from MiSeq were deposited in the DDBJ database under accession numbers DRA006847 and DRA008653.
